# Host specificity pattern and chemical deception in a social parasite of ants

**DOI:** 10.1038/s41598-018-38172-4

**Published:** 2019-02-07

**Authors:** Luca Pietro Casacci, Karsten Schönrogge, Jeremy Ambler Thomas, Emilio Balletto, Simona Bonelli, Francesca Barbero

**Affiliations:** 1 Museum and Institute of Zoology, Polish Academy of Sciences, Warsaw, 00-679 Poland; 2 Department of Life Sciences and Systems Biology, University of Turin, Turin, 10123 Italy; 3 Centre for Ecology and Hydrology, Wallingford, OX10 8BB United Kingdom; 4 Department of Zoology, University of Oxford, Oxford, OX1 3PS United Kingdom

## Abstract

In natural ecosystems, relationships between organisms are often characterised by high levels of complexity, where vulnerabilities in multi-trophic systems are difficult to identify, yet variation in specific community modules can be traceable. Within the complex community interactions, we can shed new light on dynamics by which co-evolutionary outcomes can inform science-led conservation. Here we assessed host-ant use in six populations of the butterfly *Phengaris* (=*Maculinea*) *rebeli*, an obligate social parasite of *Myrmica* ants and a model system in evolutionary and conservation ecology. Starting from the initial distribution of eggs, we estimated the survival of the parasite in the wild in nests of seven *Myrmica* ant species, and analysed the chemical cues evolved by the parasites to subvert its host defences. We found local variations in host specificity that are consistent with similarities found in the chemical profiles of hosts and parasites on different sites. At some sites, only one ant species is successfully exploited; at others, multiple-host populations are used. Understanding how stable or adaptable these associations are is essential knowledge when devising conservation measures to maintain keystone species of ant and locally adapted populations of *Phengaris* butterfly species, which are rare, threatened and a high priority for conservation worldwide.

## Introduction

Although many generalist insect species can respond rapidly to environmental changes^1,2^, closely-coupled assemblages of interacting specialists are often more vulnerable, because survival may depend upon the maintenance of obligate co-adaptations or interactions within the community^3^. An estimated ~100000 species of insect are myrmecophiles that interact with ants^4^; most being facultative, often mutualistic, and displaying similar diffuse co-evolutionary patterns. About 10000 species are obligatory myrmecophiles, including many with antagonistic interactions in the form of social parasitism, where we expect to find tighter co-evolution between parasite and host, potentially in a geographical mosaic^5,6^. The European *Phengaris* (=*Maculinea*) (*P. arion, P. teleius, P. nausithous, P. alcon, P. rebeli –* we considered the two latter taxa as separate following^7^) are already the most studied ant-parasitic butterflies, and have become a model system in evolutionary and conservation ecology^8^. *P. rebeli* adults, the focal species of this study, are on the wing from late June to mid-July and females oviposit on *Gentiana cruciata*. About four days after egg-laying, *P. rebeli* larvae hatch and feed for 10–14 days on their specific food plant. After the third moult, larvae leave the plant and drop to the ground where they are found by a *Myrmica* worker and carried into an ant nest^9^. The adoption of caterpillars by ants is primarily mediated by chemical deception^6,10–13^. As demonstrated for *P. rebeli* in South-Western Europe, pre-adoption caterpillars synthesise a simple mixture of surface hydrocarbons that weakly mimics those of *Myrmica* species in general, but has the closest match to the hydrocarbon signature of its host ant *M. schencki*^14^. Yet the low level of chemical similarity, coupled with poor discrimination by foraging workers, means that *P. rebeli* pre-adoption caterpillars are retrieved into nests by workers of any foraging *Myrmica* species that happen to encounter them^10,11,14,15^. Once inside ant colonies, the mimetic cocktail of hydrocarbons synthesised by caterpillars becomes more complex^11,15,16^, which, aided by acoustical mimicry^17–19^, not only enables the parasites to integrate closely with the societies of their primary ant host species but also identifies them as intruders to “non-host” species, where, in due course, either all or the large majority of individuals are killed or ejected by the more discriminatory nurse-workers^11,15,20,21^. Inside host ant nests, *Phengaris* larvae are fed mainly by the regurgitations of nurse ants, gaining about the 98% of their final body mass^4^ over a period of either 11 or 23 months^4^, the result of a developmental polymorphism that exists in some populations. Full-grown butterfly larvae pupate in the upper chambers of the ant nest and emerge unharmed as adults via the colony’s galleries.

More recent field studies have shown that an early finding, that each *Phengaris* species was an obligate parasite of a single and different *Myrmica* species, was oversimplified at a continental scale^22^. *P. rebeli* is now recorded from eight different *Myrmica* species in separate parts of its range in Europe^23 and references therein^. However, while the existence of distinct host races within *Phengaris* species is well established^15^, it has been shown that *P. alcon* and *P. rebeli*, in particular, tend to be specific to a single *Myrmica* host species at population and usually regional scales, even though several potential *Myrmica* species co-exist in abundance on the same sites and largely overlap in their distributions^21^. More recently, “multiple-host using populations” have been reported for certain *P. alcon* and *P. teleius* populations in Denmark^24^, Poland^25^ and Hungary^26^. Because non-host *Myrmica* colonies are known to tolerate *Phengaris* parasites if the colony is well provisioned, it remains unclear whether these comparatively rare instances represent random occasions driven by processes not dependent on the chemical mimicry the parasites have evolved or whether host associations differ fundamentally in these populations^21^.

To distinguish the two possibilities, we estimated the survival rates, rather than study the occurrence, of *Phengaris rebeli* caterpillars in *Myrmica* host colonies at six sites in Italy, and analysed similarities in the cuticular hydrocarbon signatures among host ants and parasites on which the chemical mimicry is based. We suggest that where the estimated survival rates do not differ between multiple *Myrmica* host species, the generalist host use is based on host-parasite interactions rather than environmental factors. How tight the relationships between the host ant and parasite are is central not only to understanding the evolution of widespread associations between butterflies and ants, but also for the survival and conservation of *Phengaris* spp., especially when faced with additional threats such as rapid climate change that necessitate more intrusive land management^27^.

## Results

### *Myrmica* communities

**Figure 1:**
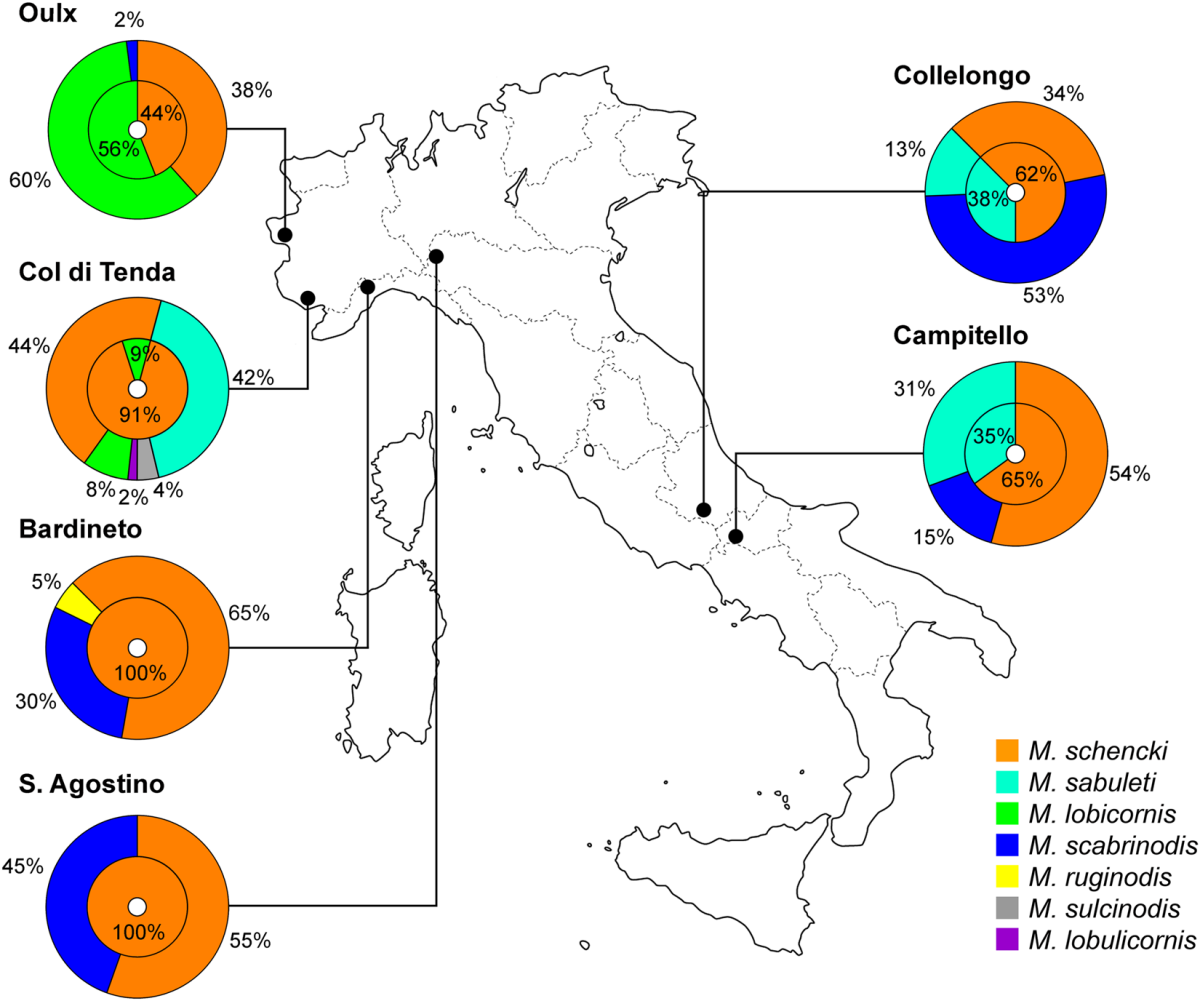
For each site, the outer ring shows the proportion of *P. rebeli* eggs occurring in the foraging range of different *Myrmica* species in summer. The inner circles show the proportion of full-grown larvae or pupae from *Myrmica* colonies in the following spring.

We located and excavated 186 *Myrmica* nests within 2 m (=foraging range) of *G. cruciata* plants in the 6 sites (Fig. 1). Each site supported 2 to 5 species of *Myrmica* (Fig. 1; Table S1), with *M. schencki* present at all sites and overall the commonest species present (43% of all colonies found). *M. scabrinodis* was found at all sites except for Col di Tenda. In contrast, *M. lobulicornis* and *M. sulcinodis* were found only at Col di Tenda, and just four nests of *M. ruginodis* were found at Bardineto. At the two southernmost sites, Collelongo and Campitello, we found the same *Myrmica* community of *M. schencki, M. sabuleti* and *M. scabrinodis*. At the northern alpine site (Oulx), we also found colonies of *M. lobicornis* as well as *M. schencki* and *M. scabrinodis*.

### *Parasitism by* Phengaris rebeli

*P. rebeli* females laid eggs singly or in small groups on flower buds and the uppermost leaves of *Gentiana cruciata* plants. The average number of eggs per plant varied greatly across the Italian peninsula; the highest average (±SD) values were found at the northern sites of Oulx (13.4 ± 10.7) and Bardineto (13.4 ± 7.7), the lowest at the southern site of Collelongo (3.2 ± 2.0) (Table S1).

At all sites apart from S. Agostino, the distribution of eggs laid on *G. cruciata* growing in the territories of different *Myrmica* species differed significantly from the distribution of all gentians available for oviposition to *P. rebeli*: (*X*^2^_site,df_; *X*^2^_S.Agostino,2_ = 1.52, p = 0.22; *X*^2^_Oulx,2_ = 155.06, p < 0.001; *X*^2^_Bardineto,2_ = 32.44, p < 0.001; *X*^2^_Tenda,4_ = 16.01, p = 0.003; *X*^2^_Campitello,2_ = 36.83, p < 0.001; *X*^2^_Collelongo,2_ = 12.24, p = 0.002). At Bardineto, Campitello and Col di Tenda, a large proportion of eggs was associated with the presence of *M. schencki*, while at Oulx and Collelongo a disproportionate number of eggs was laid in the foraging areas of *M. lobicornis* and *M. scabrinodis* colonies, respectively (Fig. 1). Since previous studies showed no differential mortality of the eggs or early larval instars on *G. cruciata* growing in the niches of different *Myrmica* species, nor any bias in the discovery and retrieval of wild final-instar larvae from beneath food plants, *P. rebeli* egg distributions may be taken as equating to the proportion of young larvae entering nests of the different ant species^28,29 see the appendix^.

The following summer we found a total of 137 *Phengaris* individuals in their final stages of growth (full-grown larvae or pupae) in 52 (28%) of the 186 *Myrmica* ant nests excavated, with site infestation levels ranging from 10% (S. Agostino, Collelongo) to 43% (Campitello). In contrast to the races of *P. rebeli* studied in the Pyrenees and French Hautes-Alps^30^, only one biennial *P. rebeli* larva was found (within one *M. schencki* colony at Col di Tenda) indicating that the incidence of polymorphic larvae in Italy is extremely low.

The mean (±SE) number of *Phengaris* full-grown larvae or pupae per infested nest was 2.6 ± 1.8, ranging from 1 to 8 individuals per colony. *P. rebeli* full-grown larvae or pupae were found only in *M. schencki* nests at Bardineto and S. Agostino (Northern Apennines), while at Col di Tenda and Oulx, we found the parasite inside the nests of both *M. schencki* and *M. lobicornis*. In the southernmost sites of Collelongo and Campitello, both *M. sabuleti* and *M. schencki* colonies were exploited (Fig. 1; Table S1).

**Figure 2:**
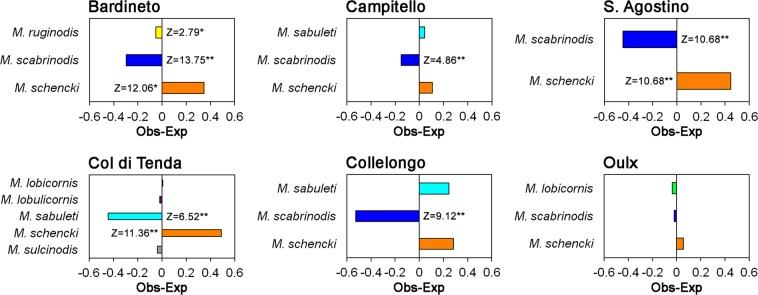
Differences between the expected and observed proportions of full-grown larvae or pupae of *P. rebeli* found in the nests of *Myrmica* species occurring at each site. For all sites the proportion of eggs found on gentian plants in late summer associated with each *Myrmica* species were compared to the proportion of *P. rebeli* full-grown larvae or pupae found in each *Myrmica* colony in the following spring. Z = Z-score Two-Proportion Z tests; *p < 0.05; **p < 0.01.

Results of the Two-Proportion Z tests showed that at Bardineto, Col di Tenda and S. Agostino *P. rebeli* survived significantly better in *M. schencki* colonies (Fig. 2). At Campitello, we found that estimated survival rates in nests of *M. sabuleti* and *M. schencki* were similar, but *P*. *rebeli* experienced significantly higher mortality when living with *M. scabrinodis* (Fig. 2). A similar pattern was found at Collelongo, although we found only 3 infested nests. *P. rebeli* showed slightly higher estimated survival in *M. schencki* nests than was expected from the distribution of eggs at Oulx, but the change in proportions was not significant with any of the *Myrmica* species present.

### Patterns in chemical profiles

MDS ordinations of the 75 samples based on the Euclidean distances calculated on relative abundances of 46 CHCs produced a good discrimination between the five ant species and *P. rebeli* larvae (ANOSIM: R = 0.948, p = 0.001; 2D STRESS value = 0.07).

Pooling the samples from all localities indicated that the averaged chemical profiles of *P. rebeli* larvae were closer to those of *M. schencki* (Mean ± SE = 44.86 ± 0.47) than to other ant species (Mann–Whitney U test: p < 0.001 for all comparisons; Table S2). A slightly less similar cuticular composition from *P. rebeli* caterpillars was found for *M. sabuleti* from Campitello (55.71 ± 0.52) and for *M. lobicornis* workers (58.32 ± 0.35), but the highest chemical distance was invariably assessed for *M. scabrinodis* ant profiles (81.66 ± 0.25).

**Figure 3:**
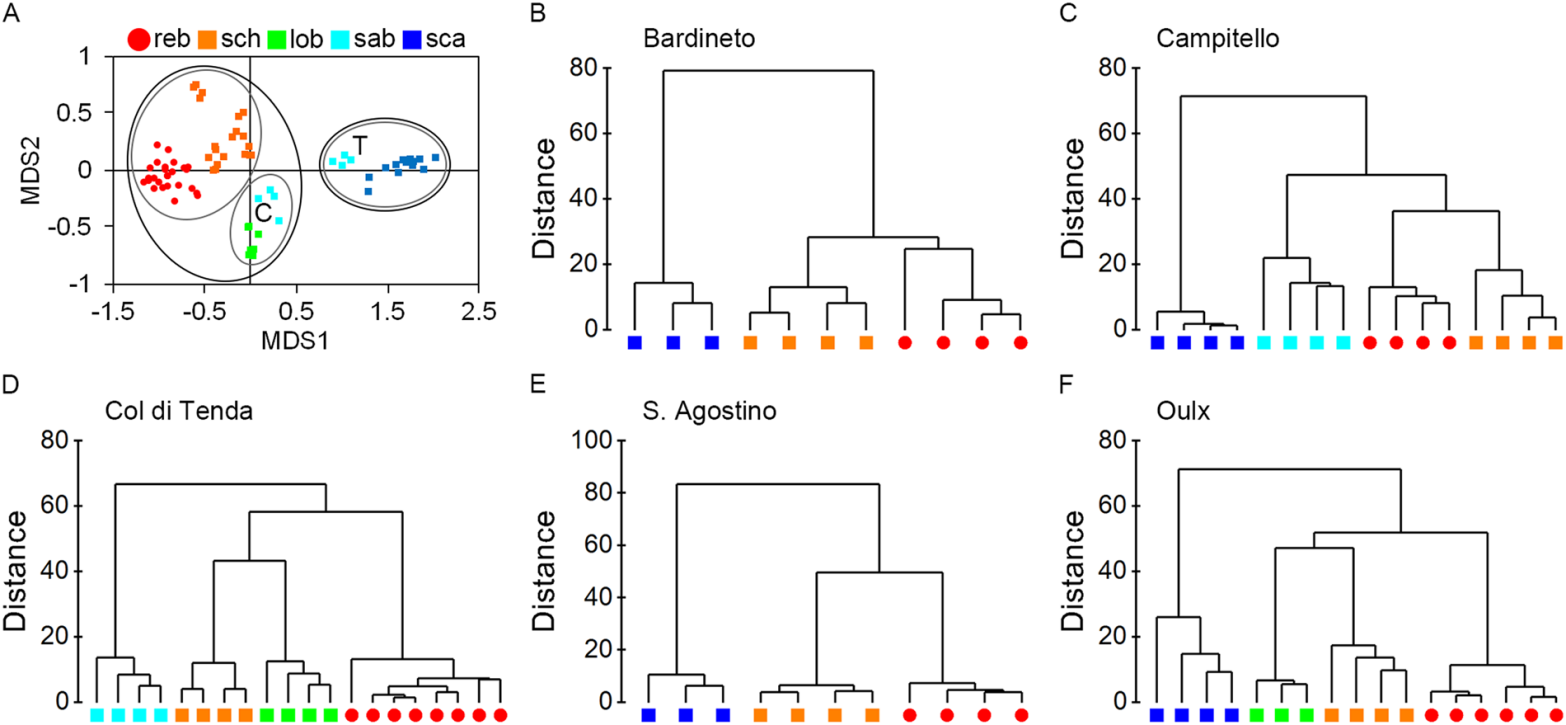
(**A**) Nonmetric multidimensional scaling (NMDS) plot of the cuticular hydrocarbon profiles across all study sites of *Myrmica* species: sch = *M. schencki; sab* = *M. sabuleti, lob* = *M. lobicornis* and *scab* = *M. scabrinodis*, and reb = *P. rebeli* pre-adoption caterpillars. Circles delimit clusters of samples with 50 (grey lines) and 70 (black lines) average Euclidean distances. C, indicates *M. sabuleti* samples from Campitello; T, indicates *M. sabuleti* samples from Col di Tenda. 2D Stress = 0.07. (**B**–**F**) Dendrograms generated by the cluster analysis of Euclidean distances based on hydrocarbon chemical profiles of the four ant species *M. schencki*, *M. sabuleti*, *M. lobicornis* and *M. scabrinodis* and *P. rebeli* pre-adoption caterpillars which had never been exposed to ants. Colours in the legend of the NMDS plot also refer to the dendrograms.

These findings are reported in the MDS plot where results of CLUSTER analysis on all data are superimposed. The first level (average Euclidean distance = 50, Fig. 3A) highlights the similarity between *M. schencki* and *P. rebeli* profiles, which cluster together, and pool *M. lobicornis* and *M. sabuleti* samples from Campitello in a second cluster. The second Euclidean distance level (average Euclidean distance = 70, Fig. 3A) separated *P. rebeli* and the three *Myrmica* species exploited as hosts (*M. schencki*, *M. lobicornis*, *M. sabuleti* from Campitello) from non-host species (*M. sabuleti* from Col di Tenda and *M. scabrinodis* from any site).

It is noteworthy that the CHCs of *M. sabuleti* from Campitello and Col di Tenda differed significantly (ANOSIM: R = 1, p = 0.029). Those from Campitello were more similar to the profile of *P. rebeli* larvae, while *M. sabuleti* workers at Col di Tenda showed substantial chemical distances (71.56 ± 0.40) from their parasite’s profile (Mann–Whitney U test: U = −337.356, df = 1, p < 0.001), consistent with the 100% mortality of *P. rebeli* in *M. sabuleti* nests on the latter site (Fig. 1).

In the Cluster analyses (Fig. 3B,E), Bardineto and S. Agostino show a similar pattern, with *P. rebeli* closer to *M. schencki* than to *M. scabrinodis* (Mann–Whitney U test: p < 0.001), which was never exploited as host. The butterfly exploited both *M. sabuleti* and *M. schencki* at Campitello (Fig. 3C), and although *P. rebeli*’s CHC profile was closer to that of *M. schencki* (36.35 ± 1.38) than to *M. sabuleti* (47.93 ± 1.09), this difference was not significant (Mann–Whitney U test: U = 20.750, df = 1, p = 0.092). In the Oulx population (Fig. 3F), the lowest distances were found between *P. rebeli* larvae and *M. schencki* worker ants (49.62 ± 0.81), with the chemical distance of caterpillars to *M. lobicornis* samples (55.22 ± 0.65) slightly greater than to *M. schencki* (Mann–Whitney U test: U = 19.375, df = 1, p = 0.115). At Col di Tenda (Fig. 3D), the cuticular profile of *P. rebeli* caterpillars was chemically closer to *M. schencki* (55.82 ± 0.52) than to *M. lobicornis* (60.85 ± 0.71) (Mann–Whitney U test: U = 36.125, df = 1, p = 0.009) or to *M. sabuleti* workers (74.83 ± 0.47) (Mann–Whitney U test: U = 82.813, df = 1, p < 0.001).

Within each species of *Myrmica*, workers taken from the same site were chemically more similar to each other than to specimens of the same species collected at different sites. This difference was statistically significant for *M. schencki* (ANOSIM: R = 0.974, p = 0.001), *M. sabuleti* (ANOSIM: R = 1, p = 0.029) and *M. lobicornis* (ANOSIM: R = 1, p = 0.029). In the case of *M. scabrinodis*, the global ANOSIM test was also significant (ANOSIM: R = 0.209, p = 0.038), but none of the pairwise comparisons between the CHC profiles of *M. scabrinodis* workers from different sites were significant (ANOSIM: p > 0.057). Even the *P. rebeli* caterpillars from different sites could be distinguished by their CHC profiles (ANOSIM: R = 0.715, p = 0.001), apart from those larvae collected in Bardineto and S. Agostino, which were chemically similar (ANOSIM: R = 0.490, p = 0.057). In general, the average chemical distance between the CHC profiles of *M. rebeli* caterpillars and *Myrmica* workers explained the estimated survival of *P. rebeli* full-grown larvae or pupae within *Myrmica* colonies (*GLMM*: estimate ± SE = −0.078 ± 0.029, z = −2.733, p = 0.006; R^2^_m_ = 0.287; R^2^_c_ = 0.420), meaning that when the chemical profiles of *P. rebeli* pre-adoption larvae were more similar to a certain *Myrmica* species, the likelihood of finding successful full-grown larvae or pupae within ant colonies was higher.

### Myrmica *niches at* Phengaris rebeli *sites*

**Figure 4:**
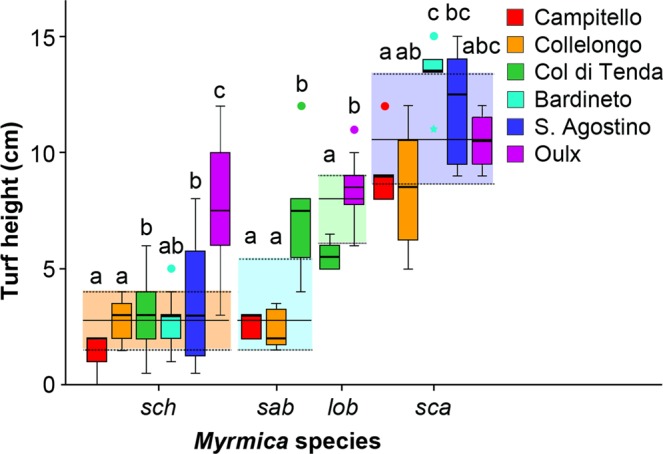
The turf height within 2 m of the most abundant *Myrmica* species (*M. schencki, M. sabuleti, M. lobicornis*, *M. scabrinodis*) at the study sites. The boxplots show the niches for each species within each site (thick line = median turf height, box = first and third quartiles, whiskers = minimum and maximum values or 1.5 interquartile range, dots and star = outliers). Colour panels show the range between the first and third quartile of turf heights for each *Myrmica* species and the lines represent the median values. Different letters indicate statistically significant categories based on Mann–Whitney U tests (Table S3) for each species within each site.

Our results confirm that each *Myrmica* species is associated with a different turf height (Kruskal–Wallis one-way ANOVA: H = 92.071, d = 3, p < 0.001; Fig. 4). *M. schencki* inhabited shorter turf (Mean ± SE: 3.43 ± 0.28 cm) than *M. lobicornis* (7.8 ± 0.39 cm) or *M. scabrinodis* (Mann–Whitney U test: p < 0.001 for both comparisons), the last being associated with the tallest turf (10.47 ± 0.46 cm) at all sites. *M. sabuleti* at the southern populations occupied the same niche as *M. schencki* (Mann–Whitney U test: U = 1.059, df = 1, p = 0.303), while at Col di Tenda the height of grass surrounding *M. sabuleti* colonies was significantly higher (Mann–Whitney U test: Campitello, U = 16.303, df = 1, p < 0.001; Collelongo, U = 18.313, df = 1, p < 0.001). At Oulx we did not find a significant difference in the turf height among the three *Myrmica* species (*M. schencki*, *M. lobicornis* and *M. scabrinodis*) (Kruskal–Wallis one-way ANOVA: H = 5.060, df = 2, p = 0.080) but, surprisingly, the height of the grass surrounding *M. schencki* colonies was higher than on other sites (Mann–Whitney U test: p < 0.004; Table S3).

## Discussion

Our results, based on the estimated survival of parasitic larvae or pupae, reveal that the *M. schencki* adapted race of *P. rebeli* inhabits most sites across the Italian peninsula, and that the parasite suffered disproportionately higher or total mortalities when adopted into nests of other *Myrmica* species, which can be classed, sensu Thomas *et al*.^21^, respectively as secondary or non-host *Myrmica* species. *M. schencki* was the commonest species and was used as host at all sites, but in Campitello and Collelongo, the parasite was reared with equally success in nests of *M. sabuleti*, suggesting the existence of communities with two primary hosts (multiple-host populations). Communities with more than two primary host species were never observed.

At Bardineto and S. Agostino, *P. rebeli* survived exclusively with *M. schencki* and can be ascribed to single-host populations. But if, as in most studies, we had sampled only for the presence of the parasite as full-grown larvae or pupae, the populations at Col di Tenda and Oulx would have been reported as exploiting multiple hosts; instead, after factoring in the initial distribution of eggs on each site, our results indicate the presence of a primary host, *M. schencki* and a secondary host (sensu Thomas *et al*.^21^), *M. lobicornis*, at least at Col di Tenda. The pattern of survival rates at Oulx must be interpreted with caution because the differences are not statistically significant. However, the large number (28) of *P. rebeli* full-grown larvae or pupae found in the nests of *M. lobicornis*, together with the results found in the other alpine site (Col di Tenda), suggests that this species might be considered a sub-optimal “secondary” host (sensu Thomas *et al*.^21^).

It is notable that no surviving full-grown larvae or pupae were recorded from *M. scabrinodis* colonies, even though at Collelongo and San Agostino 53% and 45% of *P. rebeli* eggs were respectively laid beside its nests, and a similar proportion of larvae can be assumed^4,28,29^ to have been adopted into its colonies. In contrast, *M. scabrinodis* is the sole host exploited by *P. rebeli*’s sibling species, *P. alcon*, across most of Europe.

We also confirmed and quantified distinct differences in the optimum turf structure inhabited by the various *Myrmica* species at these latitudes and altitudes, providing essential knowledge for the future grazing management and conservation of this threatened butterfly. Only at Oulx was there an apparent absence of turf height preference by different *Myrmica* species. This is possibly explained by the relatively homogeneous micro-topography at Oulx and by the fact that the grassland was rarely grazed in recent years, and is at a transitional stage towards taller turf. We suspect that *M. schencki* is persisting sub-optimally before being succeeded by *M. lobicornis*, which is adapted to colder microclimatic niches^31^ and towards which *P. rebeli* may already be adapting its mimetic semio-chemicals.

To our knowledge, this is the first field-study measuring a direct correlation between the CHC similarity of caterpillars and workers and the estimated survival rate of a social parasite within the ant colonies in populations where multiple host species are exploited. The chemical similarity between pre-adoption *P. rebeli* larvae and *Myrmica* ants explained a significant proportion (around the 30%) of the variation in the estimated parasite survival (R^2^_m_ = 0.287). Random factors (ant species nested within sampling sites) increased the explanatory power (R^2^_c_ = 0.420) suggesting that the parasite survival within the nest can be linked to other ant species traits and butterfly adaptations (e.g. vibroacoustic emissions). Overall, *P. rebeli* pre-adoption CHC profiles from all available populations were most similar to *M. schencki* workers compared to those of other *Myrmica* species^10^.

Only at Oulx did *P. rebeli* pre-adoption caterpillars show an analogous level of chemical similarity to *M. lobicornis* compared to *M. schencki*, explaining the high number of full-grown larvae or pupae found within *M. lobicornis* colonies. Nevertheless *P. rebeli* larvae may lack perfect adaptation to this host during the “full integration” phase when additional mimetic chemicals are secreted once larvae are underground^11,15^, resulting in a lower observed proportion of full-grown larvae or pupae than expected compared with a fully-adapted local race. This is consistent with laboratory experiments in which *P. rebeli* caterpillars were successfully adopted by various *Myrmica* species but subsequently experienced severe mortality in all except host-species’ colonies^15^, especially when stressed^20^.

It is also important to note that CHC profiles for each *Myrmica* species, with the exception of *M. sabuleti*, resulted in single clusters in Fig. 3A. In contrast, CHCs of *M. sabuleti* from Col di Tenda and Campitello differed significantly, and were more similar to *P. rebeli* CHC profiles at Campitello where *M. sabuleti* was identified as one of the primary host species. The differences in the CHC profiles among the *M. sabuleti* workers from Campitello and Col di Tenda could possibly also result from the two ant populations occupying different niches^32^ or belonging to an as yet unidentified cryptic species (a regular occurrence in *Myrmica*)^31^. Data collected on the turf height indicate that at the cool alpine site (Col di Tenda), *M. sabuleti* colonies occur in areas where the turf is taller than the patches occupied by the southernmost populations. If there was a single temperature optimum, we would expect them to occupy higher turf (=cooler soil surface temperatures) at warmer sites and lower turf areas at cooler sites. It is also worth noting that the turf height surrounding *M. sabuleti* nests at the southern sites (Campitello and Collelongo) was not significantly different from that surrounding *M. schencki* nests, so that they share similar temperature niches and probable diets, which might also affect the CHC profiles and the differences between the *M. sabuleti* populations^33^.

Evidence of regional switches in host specificity in certain *Phengaris* species is increasing^15^. In Europe, *P. rebeli* was described as having two forms, one adapted exclusively to exploit *M*. *schencki* in Western Europe (Pyrenees, Haute-Alpes), the other dependent on *M*. *sabuleti* in Central-Northern Europe (primarily Poland)^4,15,21^, even though the majority of caterpillars are adopted by *M. sabuleti* in Spain, and *vice versa* in Poland, due to the major severe depression of host colonies inflicted by the rapacious caterpillars on occupied sites^34^. In each region, the CHC profiles synthesised (as opposed to acquired) by *P. rebeli* from Polish and Spanish populations differed prior to adoption and, especially, after 4–6 days with ants, and explained the different survival with different *Myrmica* species^15^. The difference in the CHC profile of the two populations of *M. sabuleti* may reflect the different niche utilisation and probably diet affecting the ant profiles or the existence of separate ant clades, or cryptic species, as reported by Ueda and colleagues^35^ for *M. kotokui* ants hosting *P. teleius* and *P. arionides* in Japan. Further genetic analyses on Italian ant populations are needed.

Rather than being a “*sabuleti* type” (as exists in Poland^15^), the high estimated survival of *P. rebeli* in nests of both *M. schencki* and *M. sabuleti* suggests that the population at Campitello and Collelongo are genuinely more generalist^24–26^. Multiple-host using *P. rebeli* at Campitello and Collelongo suggests that, while rare, there are some populations where these social parasites are less specialised^36–38^ than reported from the vast majority of populations studied in the past^22^. An alternative explanation could be that we failed to distinguish between two differentiated, specialist host races that live sympatrically on rare occasions but are otherwise cryptic, comparable to the co-existence on many sites of the non-cryptic *Phengaris* species, *P. teleius* and *P. nausithous*, which share the same food plant but generally exploit different species of *Myrmica*.

How different host races in *Phengaris* could have arisen is poorly understood. It has been suggested that European populations became isolated during glaciations in refuges in the Southern Alps, Western Hungary and South-Eastern Europe^39^, where different host associations may have evolved in isolation^40^. Re-colonising the continent, the different host types could have given rise to the specialised populations in Western^22,31^ and East-Central Europe^36–38^. Within such a scenario, the Alps can be regarded as a geographical and genetic barrier for butterflies, separating Italian and French populations. This could explain the lack of a developmental polymorphism on the Italian side in contrast to the strong evidence of this phenomenon in French populations^30,41^.

Alternatively, populations might experience rare host switches during the history of the species, which were too recent to be detected in their mitochondrial DNA^42^. The distribution of the host types over large, non-overlapping regions of Central and Northern Europe suggest that these are rare events, because frequent host shifts would be expected to produce geographic mosaics^5^. The fact that we do not observe multiple-host use, i.e. transient populations, often, suggests transitions are quick. A mathematical model based on the *Phengaris* system also suggests that multiple-host use can arise in both a transient or stable state, while single host use is the more likely outcome across a wide range of the parameter space^43^. A separate modelling approach also suggested that multiple-host use is more likely on sites where the similarity between the chemical profiles of distinct host ant species is high. This scenario enables the successful exploitation and deception of the hosts, without requiring a super-specialisation of the parasite that instead can evolve an intermediate CHC profile^44,45^.

Apart from microbial parasites, there are rather few host-parasite systems where the variation in the parasite phenotype can be related directly to survival with one or more hosts. Another example where this is possible and has been exploited in numerous studies involves avian brood parasites such as cuckoos^46^. Yet there is enormous interest in a better understanding of species interactions and their degree of specificity, not least because range changes under climate change may cause some species interaction pairs break, while new ones might form^47^, which clearly would be difficult for extreme specialists^48^. Generalism is seen as a costly parasite trait, and apparently generalist species are increasingly found to represent a complex of more specialised cryptic species^49,50^. Yet our study suggests even in species that are specialist throughout most of their ranges, more generalist populations exist^47,51^; although they may be difficult to identify. If this was true in other specialist parasite species as well, it suggests that they are more resilient to range changes at species level than previously thought and might possess more adaptive potential if selection for generalism predominates. More immediately, however, understanding the host-associations and how stable or adaptable they are, makes a major contribution to the conservation effort of these high priority butterfly species^52,53^. Identifying multiple-host-use populations could provide a potentially important genetic resource, but more importantly an opportunity to study what triggers host switches and what happens during transition periods.

## Materials and Methods

### Myrmica communities sampling

Egg counts were performed to estimate the relative number of *P. rebeli* caterpillars that each *Myrmica* ant colony and species received^22,28,29^. At each site, by the end of July, 30 individuals of the host plant *Gentiana cruciata* were selected at random, marked and the number of *P. rebeli* eggs was recorded on each plant.

Ant baits were placed at the base of each selected gentian to verify if the plant was visited by *Myrmica* foragers. A visual search of *Myrmica* nests was also performed in a 2 m radius from the food plant, which approximates to the foraging area for workers of these *Myrmica* species^31^. In most cases, only one nest was found in the area around each gentian, therefore all the eggs laid on the plant were referred to a *Myrmica* sp. nest. Six plants grew within the foraging range of two distinct colonies. When this occurred, they belonged to the same species and we halved the number of counted eggs and attributed them to each nest. All nests were georeferenced and marked in order to identify them the following spring. Since all *Myrmica* species present at the sites adopt *P. rebeli* caterpillars with equal alacrity^4^, and because initial larval survival on gentians does not differ on plants growing in different ant niches^28,29^, the number of eggs on plants reflects the frequency at which *P. rebeli* caterpillars are carried into nests of different *Myrmica* species in late summer^54^. The following April to June, we pinpointed again all *Myrmica* nests within a 2 m radius around each of the 30 *G. cruciata* plants at every site. No ant species substitution occurred, and each nest was identified and located. Nests were excavated and examined for the presence and abundance of *P. rebeli* full-grown larvae or pupae. After the excavation, the ground and vegetation were restored to as close to the original conditions as possible.

*Myrmica* species were identified first in the field and a sample of ten workers was collected and preserved for further inspection in the laboratory using keys by Czechowski *et al*.^55^. The *Myrmica* species identified at each site are listed in Table S1. Observed egg numbers per plant were compared with the expected values calculated as the average number of eggs per plant multiplied by the number of gentians overlapping with various *Myrmica* species, using Chi square test.

We used the proportion of eggs associated with nests of each *Myrmica* species and compared them to the proportion of *P. rebeli* full-grown larvae or pupae found inside nests of those species in the following spring, providing an estimate of relative survival with each *Myrmica* species present at each site. We adopt the term “estimated survival” throughout the text to clarify that the survival was not directly measured as in laboratory experiments. Under a Null model that the parasite survives equally well with all *Myrmica* species, the proportion of eggs associated with each species provides an expectation for the same proportions to be preserved for the full-grown larvae or pupae the following summer. Differences between expected and observed proportions of *P. rebeli* full-grown larvae or pupae were analysed using Two-Proportion Z tests. Where observed proportions at the full-grown larvae or pupae were smaller than expected, the respective *Myrmica* species was regarded as “non host”, i.e. significantly higher mortality had occurred inside the nest. Similarly, *Myrmica* species were regarded as hosts when observed proportions of *P. rebeli* full-grown larvae or pupae were greater than expected. When the estimated survival was higher than expected in more than one species we distinguish two scenarios: (i) if this difference was statistically significant only in one of the two species, the latter was considered as the “primary host” and the other as the “secondary host” sensu Thomas and colleagues^21^. Overall these populations are defined as single-host populations; (ii) if the observed proportions of *P. rebeli* full-grown larvae or pupae did not differ between the two hosts, both were considered “primary” and the population was described as “multiple-host”.

### Chemical analysis

*Phengaris* caterpillars infiltrate *Myrmica* colonies by mimicking the cuticular hydrocarbons (CHCs) used by the ants for nestmate recognition^6,10,11,15,56^. Here, we analysed the CHCs of preadoption caterpillars from five study sites and compared them with the chemical signatures of the *Myrmica* worker ants available for exploitation.

At Bardineto, Campitello, Col di Tenda, Oulx and S. Agostino we obtained *P. rebeli* caterpillars by collecting gentians bearing visible eggs. Samples collected in Collelongo were lost before chemical analysis. A plant was only collected if >5 eggs were visible and if there was at least one other plant with a larger number of eggs within a 50 cm range, a strategy that limited the number of samples but minimise the impact on this endangered butterfly. We also collected colonies of every *Myrmica* species found.

Surface hydrocarbons were extracted from five pre-adoption caterpillars per sample within six hours of leaving their food plant and before any contact with ants. Caterpillars were transferred into a clean glass vial and extracted by submerging them under 200 μl hexane for 20 min. The hexane was decanted and evaporated under N_2_ stream until analysis. Similarly, five ant workers from each colony were extracted. Samples were analysed by GC/MS using equipment and methods described by Schönrogge *et al*.^11^.

Chromatograms were analysed using MSD ChemStation E.02.01.117 (Agilent Technologies) and the area under each peak identified by its ECL (Equivalent Chain Length) index was expressed as the proportion of the sum over the area of all peaks in the chromatogram while mass spectra were analysed by comparing fragmentation patterns^57^. *P. rebeli* caterpillars and ant species differences within species between sites were compared using Euclidean distances. Samples from all sites were compared using multivariate and nonparametric multidimensional scaling (NMDS) on Euclidean distances between samples. These were analysed further by Hierarchical Cluster Analysis (CA; with group average cluster mode) and the results were combined with the NMDS plots to illustrate sample groups of particular similarity. For each study site Hierarchical Cluster analyses were performed on Euclidean distances using unweighted pair-group average (UPGMA) algorithms and dendrograms obtained. Pairwise differences between species and treatments were assessed using an analysis of similarities (ANOSIM)^58^ and the significance of group separation according to their Euclidean distances was tested by Kruskal–Wallis one-way ANOVA and Mann–Whitney U test for pairwise comparisons, having previously confirmed normality and homogeneity of variance of the data. Benjamini–Hochberg procedure was used to control for false discovery rate (FDR = 7.5%) in multiple tests. To examine the effect of *P. rebeli* chemical mimicry on the estimated survival of parasitic larvae, a Generalized Linear Mixed Model was computed (binomial error term, log-link function) using the glmer function in the R package lme4^59^. The response variable, the estimated survival in ant nests, was calculated as the proportion between the number of *P. rebeli* full-grown larvae or pupae found in spring and the number of eggs counted the previous summer. Fixed explanatory terms were the average chemical distance between the CHC profiles of *P. rebeli* caterpillars and each *Myrmica* species found. Ant species nested within sampling sites were considered random factors. We computed marginal and conditional R-squared values^60^, describing variance explained by fixed effect only, and by fixed and random effects combined. Both values were calculated using the R package ‘MuMIn’^61^. All statistics were carried out with PRIMER 6 β (PRIMER-E, Plymouth, UK), SPSS® package ver. 24 and the software R version 3.4.3.

### Myrmica *niches on* Phengaris rebeli *sites*

Turf height is correlated with soil temperature and *Myrmica* ants tend to occupy specific microclimatic niches which can vary depending on the latitude^31,62^.

Vegetation height was measured at the entrance of each excavated nest. Kruskal–Wallis one-way ANOVA followed by Mann–Whitney U test were performed with SPSS® package ver. 24 to determine significant differences among turf height in the surroundings of each of the *Myrmica* species. Benjamini–Hochberg procedure was used to control for false discovery rate (FDR = 7.5%) in multiple tests. Test for normality and homogeneity of variance showed that the data were appropriate for non-parametric statistics.

## Supplementary information

Supplementary Information

## Data Availability

The datasets generated and analysed during the current study are available from the corresponding author on request.

## References

[1] Parmesan C (1999). Poleward shifts in geographical ranges of butterfly species associated with regional warming. Nature.

[2] Warren MS (2001). Rapid responses of British butterflies to opposing forces of climate and habitat change. Nature.

[3] Walther GR (2002). Ecological responses to recent climate change. Nature.

[4] Thomas, J. A., Schönrogge, K. & Elmes, G. W. In *In*se*ct* Evolu*ti*onary *Ecology* (eds M. D. E. Fellowes, G. J. Holloway, & J. Rolff) 475–514 (Royal Entomological Society, 2005).

[5] Thompson JN (2005). Coevolution: The geographic mosaic of coevolutionary arms races. Curr. Biol..

[6] Nash DR, Als TD, Maile R, Jones GR, Boomsma JJ (2008). A mosaic of chemical coevolution in a large blue butterfly. Science.

[7] Bonelli S (2018). The first red list of Italian butterflies. Insect Conserv. Divers..

[8] Thomas JA, Settele J (2004). Evolutionary biology - Butterfly mimics of ants. Nature.

[9] Thomas JA (1984). The behaviour and habitat requirements of *Maculinea nausithous* (the dusky large blue butterfly) and (the scarce large blue) in France. Biol. Conserv..

[10] Akino T, Knapp JJ, Thomas JA, Elmes GW (1999). Chemical mimicry and host specificity in the butterfly *Maculinea rebeli*, a social parasite of *Myrmica* ant colonies. Proc. R. Soc. Lond. B.

[11] Schönrogge K (2004). Changes in chemical signature and host specificity from larval retrieval to full social integration in the myrmecophilous butterfly *Maculinea rebeli*. J. Chem. Ecol..

[12] Fürst MA, Durey M, Nash DR (2012). Testing the adjustable threshold model for intruder recognition on *Myrmica* ants in the context of a social parasite. Proc. R. Soc. Lond. B.

[13] Solazzo G, Seidelmann K, Moritz RFA, Settele J (2014). Tetracosane on the cuticle of the parasitic butterfly *Phengaris* (*Maculinea*) *nausithous* triggers the first contact in the adoption process by *Myrmica rubra* foragers. Physiol. Entomol..

[14] Elmes G, Akino T, Thomas J, Clarke R, Knapp J (2002). Interspecific differences in cuticular hydrocarbon profiles of *Myrmica* ants are sufficiently consistent to explain host specificity by *Maculinea* (large blue) butterflies. Oecologia.

[15] Thomas, J. A. *et al*. Mimetic host shifts in an endangered social parasite of ants. *Proc. R. Soc. Lond. B* **280**, ARTN 20122336.10.1098/rspb.2012.2336 (2013). 10.1098/rspb.2012.2336 PMC3574407 23193127

[16] Witek M (2013). Interspecific relationships in co-occurring populations of social parasites and their host ants. Biol J Linn Soc.

[17] Barbero F (2012). *Myrmica* ants and their butterfly parasites with special focus on the acoustic communication. Psyche.

[18] Schönrogge, K., Barbero, F., Casacci, L. P., Settele, J. & Thomas, J. A. Acoustic communication within ant societies and its mimicry by mutualistic and socially parasitic myrmecophiles. *Anim. Behav*., 10.1016/j.anbehav.2016.10.031 (2016).

[19] Barbero F, Thomas JA, Bonelli S, Balletto E, Schönrogge K (2009). Queen ants make distinctive sounds that are mimicked by a butterfly social parasite. Science.

[20] Elmes GW, Wardlaw JC, Schonrogge K, Thomas JA, Clarke RT (2004). Food stress causes differential survival of socially parasitic caterpillars of *Maculinea rebeli* integrated in colonies of host and non-host *Myrmica* ant species. Entomol. Exp. Appl..

[21] Thomas JA (2005). Primary hosts, secondary hosts and ‘non-hosts’: common confusions in the interpretation of host specificity in *Maculinea* butterflies and other social parasites of ants. *Studies on the Ecology and Conservation of Butterflies in*. Europe.

[22] Thomas JA, Elmes GW, Wardlaw JC, Woyciechowski M (1989). Host specificity among *Maculinea* butterflies in *Myrmica* ant nests. Oecologia.

[23] Witek M, Barbero F, Marko B (2014). *Myrmica* ants host highly diverse parasitic communities: from social parasites to microbes. Insect. Soc..

[24] Als TD, Nash DR, Boomsma JJ (2002). Geographical variation in host-ant specificity of the parasitic butterfly *Maculinea alcon* in Denmark. Ecol. Entomol..

[25] Sielezniew M, Stankiewicz AM (2004). Simultaneous exploitation of *Myrmica vandeli* and *M. scabrinodis* (Hymenoptera: Formicidae) colonies by the endangered myrmecophilous butterfly *Maculinea* (Lepidoptera: Lycaenidae). Eur. J. Entomol..

[26] Tartally A, Varga Z (2008). Host ant use of *Maculinea teleius* in the Carpathian-Basin (Lepidoptera: Lycaenidae). Acta Zool. Hung..

[27] Hill, J. K., Griffiths, H. M. & Thomas, C. D. In Ann*ual* Revie*w* of En*tomology, Vol 56* Vol. 56 *Annual Review of Entomology* (eds Berenbaum, M. R., Carde, R. T. & Robinson, G. E.) 143–159 (2011).

[28] Hochberg, M. E., Thomas, J. A. & Elmes, G. W. A modelling study of the population dynamics of a large blue butterfly, *Maculinea rebeli*, a parasite of red ant nests. *J. Anim. Ecol*., 397–409 (1992).

[29] Hochberg, M., Clarke, R., Elmes, G. & Thomas, J. Population dynamic consequences of direct and indirect interactions involving a large blue butterfly and its plant and red ant hosts. *J. Anim. Ecol*., 375–391 (1994).

[30] Thomas JA, Elmes GW, Wardlaw JC (1998). Polymorphic growth in larvae of the butterfly *Maculinea rebeli*, a social parasite of *Myrmica* ant colonies. Proc. R. Soc. Lond. B.

[31] Elmes GW (1998). The ecology of *Myrmica* ants in relation to the conservation of *Maculinea* butterflies. J. Insect Conserv..

[32] Morrison W, Witte V (2011). Strong differences in chemical recognition cues between two closely related species of ants from the genus *Lasius* (Hymenoptera: Formicidae). J. Evol. Biol..

[33] Liang D, Silverman J (2000). “You are what you eat”: diet modifies cuticular hydrocarbons and nestmate recognition in the Argentine ant. Linepithema humile. Naturwissenschaften.

[34] Thomas JA (1997). Field evidence and model predictions of butterfly-mediated apparent competition between gentian plants and red ants. Acta Oecol..

[35] Ueda, S., Komatsu, T., Itino, T., Arai, R. & Sakamoto, H. large blue butterflies (*Phengaris* spp., Lepidoptera: Lycaenidae) in Japan. *Sci. Rep*. **6**, 10.1038/srep36364 (2016). 10.1038/srep36364 PMC5093462 27808223

[36] Steiner FM (2003). Host specificity revisited: New data on *Myrmica* host ants of the lycaenid butterfly *Maculinea rebeli*. J. Insect Conserv..

[37] Sielezniew M, Dziekanska I, Stankiewicz-Fiedurek AM (2010). Multiple host-ant use by the predatory social parasite *Phengaris* (=*Maculinea*) *arion* (Lepidoptera, Lycaenidae). J. Insect Conserv..

[38] Meyer-Hozak C (2000). Population biology of *Maculinea rebeli* (Lepidoptera: Lycaenidae) on the chalk grasslands of Eastern Westphalia (Germany) and implications for conservation. J. Insect Conserv..

[39] Schmitt T, Hewitt GM (2004). The genetic pattern of population threat and loss: a case study of butterflies. Mol. Ecol..

[40] Tartally A, Nash DR, Lengyel S, Varga Z (2008). Patterns of host ant use by sympatric populations of *Maculinea alcon* and *M*. ‘*rebeli*’ in the Carpathian Basin. Insect. Soc..

[41] Schönrogge K, Wardlaw JC, Thomas JA, Elmes GW (2000). Polymorphic growth rates in myrmecophilous insects. Proc. R. Soc. Lond. B..

[42] Patricelli D (2013). Contrasting genetic structure of rear edge and continuous range populations of a parasitic butterfly infected by Wolbachia. BMC Evol. Biol..

[43] de Assis RA (2012). A model for the evolution of parasite-host interactions based on the *Maculinea-Myrmica* system: Numerical simulations and multiple host behavior. Nonlinear Anal.-Real World Appl..

[44] de Assis RA (2017). A theory and a mathematical model for the evolution of single and multiple host behavior in a parasite-host system (*Maculinea*-*Myrmica*). Ecol. Complex., doi:doi.org/.

[45] Schlick-Steiner BC (2004). A butterfly’s chemical key to various ant forts: intersection-odour or aggregate-odour multi-host mimicry?. Naturwissenschaften.

[46] Stokke, B.G. *et al*. Characteristics determining host suitability for a generalist parasite. *Sci. Rep*. **8**, 10.1038/s41598-018-24627-1 (2018). 10.1038/s41598-018-24627-1 PMC5908913 29674671

[47] Gilman SE, Urban MC, Tewksbury J, Gilchrist GW, Holt RD (2010). A framework for community interactions under climate change. Trends Ecol. Evol..

[48] Clavel J, Julliard R, Devictor V (2011). Worldwide decline of specialist species: toward a global functional homogenization?. Front. Ecol. Environ..

[49] Nicholls JA, Schönrogge K, Preuss S, Stone GN (2018). Partitioning of herbivore hosts across time and food plants promotes diversification in the *Megastigmus dorsalis* oak gall parasitoid complex. Ecol. Evol..

[50] Bickford D (2007). Cryptic species as a window on diversity and conservation. Trends Ecol. Evol..

[51] Sasha R, Dall X, Cuthill. IC (1997). The information costs of generalism. Oikos.

[52] Mace GM (2004). The role of taxonomy in species conservation. Philos. Trans. R. Soc. Lond. Ser. B-Biol. Sci..

[53] Casacci LP, Barbero F, Balletto E (2014). The “Evolutionarily Significant Unit” concept and its applicability in biological conservation. Ital. J. Zoolog..

[54] Elmes GW, Thomas JA, Munguira ML, Fiedler K (2001). Larvae of lycaenid butterflies that parasitize ant colonies provide exceptions to normal insect growth rules. Biol. J. Linnean Soc..

[55] Czechowski, W., Radchenko, A., Czechowska, W. & Vepsalainen, K. In *Ants of Poland - with Reference to the Myrmecofauna of Europe* Vol. 4 *Fauna Poloniae-New Series* 1–496 (2012).

[56] Barbero F (2016). Cuticular Lipids as a Cross-Talk among Ants, Plants and Butterflies. Int. J. Mol. Sci..

[57] Csata, E. *et al*. Lock-picks: fungal infection facilitates the intrusion of strangers into ant colonies. *Sci. Rep*. **7**, 10.1038/srep46323 (2017). 10.1038/srep46323 PMC5389342 28402336

[58] Clarke KR (1993). Nonparametric multivariate analyses of changes in community structure. Aust. J. Ecol..

[59] Bates, D., Maechler, M., Bolker, B. & Walker, S. lme4: Linear mixed-effects models using Eigen and S4. R package version 1.0–5, http://CRAN.R-project.org/package=lme4 (2013).

[60] Bartoń, K. *MuMIn: Multi-model inference*. R package version 1.9.13, http://CRAN.R-project.org/package=MuMIn (2013).

[61] Nakagawa S, Schielzeth H (2013). A general and simple method for obtaining R2 from generalized linear mixed‐effects models. Methods Ecol. Evol..

[62] Casacci LP (2011). Habitat preferences of *Maculinea arion* and its *Myrmica* host ants: implications for habitat management in Italian Alps. J. Insect Conserv..

