# Supplementary material for: Host specificity pattern and chemical deception in a social parasite of ants
Source: Sci Rep. 2019 Feb 7;9:1619. doi: 10.1038/s41598-018-38172-4 (PMC6367357; doi:10.1038/s41598-018-38172-4)
Supplement: Supplementary file 1 — Supplementary Information [file 41598_2018_38172_MOESM1_ESM.pdf]

Host specificity pattern and chemical deception in a social parasite of ants

Luca Pietro Casacci, Karsten Schönrogge, Jeremy Ambler Thomas, Emilio Balletto, Simona Bonelli and Francesca Barbero

SUPPLEMENTARY MATERIAL

Table S1: Site specific information on location, densities of the *P. rebeli* host plant, *Gentiana cruciata*, and *Myrmica* ants. The sampling effort and the proportion of parasitism at each site are also shown. Proportions of parasitism were calculated as the ratios between the number of infested *Myrmica* nests (irrespectively of the species) and the total number of excavated nests multiplied by 100.

Location	Latitude Longitude	Density of *G. cruciata* (plant/m^2)	Density of *Myrmica* spp. (nests/m^2)	*Myrmica* spp. recorded	Number of eggs laid on plants near *Myrmica* colonies	Nests excavated	Proportion of *Myrmica* spp. in the surrounding of gentians	Infested nests	Number of *P. rebeli* full-grown larvae or pupae	Proportion of *P. rebeli* parasitism
Bardineto	N 44 11'	0.14	0.02	*My. schencki*	263	16	54%	6	25	
	E 008 0.8'			*My. ruginodis*	21	4	13%	---	---	20%
				My. scabrinodis	119	10	33%	---	---	
Campitello	N 41° 27'	0.06	0.14	*My. schencki*	108	12	34%	8	13	
	E 014° 24'			*My. sabuleti*	61	17	49%	7	7	43%
				My. scabrinodis	30	6	17%	---	---	
Col di Tenda	N 46° 20'	0.54	0.01	*My. schencki*	77	11	37%	9	20	
	E 008° 17'			*My. lobicornis*	15	5	17%	2	2	
				My. sabuleti	81	11	37%	---	---	37%
				My. sulcinodis	7	2	7%	---	---	
				My. lobulicornis	3	1	3%	---	---	
Collelongo	N 41° 51'	2.01	0.07	*My. schencki*	34	7	22%	2	5	
	E 013° 36'			*My. sabuleti*	13	8	26%	1	3	10%
				My. scabrinodis	52	16	52%	---	---	
Oulx	N 45° 01'	4.43	0.03	*My. lobicornis*	240	10	33%	10	28	
	E 006° 48'			*My. schencki*	154	14	47%	4	22	47%
				My. scabrinodis	8	6	20%	---	---	
S. Agostino	N 44° 44'	0.05	0.02	*My. schencki*	97	18	60%	3	12	10%
	E 009° 27'			*My. scabrinodis*	78	12	40%	---	---	

Table S2: Mann-Whitney U test pairwise comparisons of the Euclidean distances calculated on the relative abundance matrix of the CHCs of *P. rebeli* and *Myrmica* worker chemical profiles. Results are presented for all samples grouped together and for each site separately. Asterisks (*) denote significant p-values controlled for false discovery rate (FDR = 7.5%) by using the Benjamini–Hochberg procedure.

All samples

Sample 1 - Sample 2	Mann-Whitney U	P
P. rebeli-M. schencki vs *P. rebeli-M. sabuleti*C	239.463	0.000*
P. rebeli-M. schencki vs *P. rebeli-M. lobicornis*	319.738	0.000*
P. rebeli-M. schencki vs *P. rebeli-M. sabuleti*T	576.819	0.000*
P. rebeli-M. schencki vs *P. rebeli-M. scabrinodis*	785.741	0.000*
*P. rebeli-M. sabuleti*C vs *P. rebeli-M. lobicornis*	80.275	0.076
*P. rebeli-M. sabuleti*C vs *P. rebeli-M. sabuleti*T	-337.356	0.000*
*P. rebeli-M. sabuleti*C vs *P. rebeli-M. scabrinodis*	-546.277	0.000*
P. rebeli-M. lobicornis vs *P. rebeli-M. sabuleti*T	-257.081	0.000*
P. rebeli-M. lobicornis vs *P. rebeli-M. scabrinodis*	-466.003	0.000*
*P. rebeli-M. sabuleti*T vs *P. rebeli-M. scabrinodis*	-208.922	0.000*

Bardineto

P. rebeli-M. schencki vs *P. rebeli-M. scabrinodis*	27.000	0.000*

S. Agostino

P. rebeli-M. schencki vs *P. rebeli-M. scabrinodis*	25.500	0.000*

Campitello

P. rebeli-M. schencki vs *P. rebeli-M. sabuleti*	20.750	0.092
P. rebeli-M. schencki vs *P. rebeli-M. scabrinodis*	76.688	0.000*
P. rebeli-M. sabuleti vs *P. rebeli-M. scabrinodis*	-55.938	0.000*

Oulx

P. rebeli-M. schencki vs *P. rebeli-M. lobicornis*	19.375	0.115
P. rebeli-M. schencki vs *P. rebeli-M. scabrinodis*	68.500	0.000*
P. rebeli-M. lobicornis vs *P. rebeli-M. scabrinodis*	-49.125	0.000*

Col di Tenda

P. rebeli-M. schencki vs *P. rebeli-M. lobicornis*	36.125	0.009*
P. rebeli-M. schencki vs *P. rebeli-M. sabuleti*	82.813	0.000*
P. rebeli-M. lobicornis vs *P. rebeli-M. sabuleti*	-46.688	0.001*

Table S3: Mann-Whitney U test pairwise comparisons of the turf height surrounding the nests of the four main *Myrmica* species (*M. schencki, M. scabrinodis, M. sabuleti, M. lobicornis*). Asterisks (*) denote significant p-values controlled for false discovery rate (FDR = 7.5%) by using the Benjamini–Hochberg procedure.

M. schencki	Mann-Whitney U	P
Campitello vs Bardineto	16.279	0.068
Campitello vs Collelongo	-19.393	0.078
Campitello vs Tenda	-20.659	0.034*
Campitello vs S. Agostino	22.967	0.007*
Campitello vs Oulx	-48.950	0.000*
Bardineto vs Collelongo	-3.113	0.757
Bardineto vs Tenda	-4.380	0.613
Bardineto vs S. Agostino	6.688	0.350
Bardineto vs Oulx	-32.671	0.000*
Collelongo vs Tenda	-1.266	0.907
Collelongo vs S. Agostino	3.575	0.711
Collelongo vs Oulx	-29.557	0.007*
Tenda vs S. Agostino	2.308	0.778
Tenda vs Oulx	28.291	0.004*
S. Agostino vs Oulx	-25.983	0.002*

M. scabrinodis		
Collelongo vs Campitello	0.967	0.872
Collelongo vs Oulx	-7.442	0.256
Collelongo vs S. Agostino	13.138	0.014*
Collelongo vs Bardineto	19.567	0.000*
Campitello vs Oulx	-6.475	0.407
Campitello vs S. Agostino	12.171	0.074
Campitello vs Bardineto	18.600	0.004*
Oulx vs S. Agostino	5.696	0.435
Oulx vs Bardineto	12.125	0.083
S. Agostino vs Bardineto	-6.429	0.273

M. sabuleti		
Collelongo vs Campitello	2.013	0.637
Collelongo vs Tenda	-18.313	0.000*
Campitello vs Tenda	-16.300	0.000*

M. lobicornis		
Oulx vs Tenda	9.968	0.002*
